# The Optimization of Spacer Engineering for Capacitor-Less DRAM Based on the Dual-Gate Tunneling Transistor

**DOI:** 10.1186/s11671-018-2483-8

**Published:** 2018-03-05

**Authors:** Wei Li, Hongxia Liu, Shulong Wang, Shupeng Chen, Qianqiong Wang

**Affiliations:** Key Laboratory for Wide Band Gap Semiconductor Materials and Devices of Education, School of Microelectronics, Xidian University, Xi’an, 710071 China

**Keywords:** Tunneling FET (TFET), DRAM, Spacer engineering, Retention time

## Abstract

The DRAM based on the dual-gate tunneling FET (DGTFET) has the advantages of capacitor-less structure and high retention time. In this paper, the optimization of spacer engineering for DGTFET DRAM is systematically investigated by Silvaco-Atlas tool to further improve its performance, including the reduction of reading “0” current and extension of retention time. The simulation results show that spacers at the source and drain sides should apply the low-k and high-k dielectrics, respectively, which can enhance the reading “1” current and reduce reading “0” current. Applying this optimized spacer engineering, the DGTFET DRAM obtains the optimum performance-extremely low reading “0” current (10^−14^A/μm) and large retention time (10s), which decreases its static power consumption and dynamic refresh rate. And the low reading “0” current also enhances its current ratio (10^7^) of reading “1” to reading “0”. Furthermore, the analysis about scalability reveals its inherent shortcoming, which offers the further investigation direction for DGTFET DRAM.

## Background

With the shrink of device geometry, the 1 transistor (1T)–1 capacitor (1C) dynamic random access memory (DRAM) has encountered difficulty in scaling down, because it is difficult for capacitor to reduce its size [1–3]. The memory industry has proposed some effective methods for the packaging of higher density memory, such as new materials and novel device structures [4, 5]. The 1T DRAM with the capacitor-less structure was firstly reported in the early 90’s [6, 7], and it attracts more and more attention. In 1T DRAM, the state 1 (carrier storage) is achieved by four ways: impact ionization [8], bipolar junction transistor [9], band-to-band tunneling (BTBT) [10], and gate tunneling [11].

The tunneling field-effect transistor (TFET) based on the BTBT has been regarded as a potential alternate for MOSFET due to the higher switching ratio and extremely low off-state current [12–14]. The advantages of TFET—low off-state current and weak temperature dependence—are extraordinarily beneficial for DRAM. Especially, the low off-state current can reduce reading “0” current and static power consumption. At present, there are some groups working on the investigation of TFET DRAM [15–20]. It is reported that TFET DRAM has the low reading “0” current and high retention time (RT). Among these TFET DRAMs, the dual-gate TFET (DGTFET) DRAM is most prominent [19, 20]. In DGTFET DRAM, both the writing and reading operations are based on the BTBT. Research shows that reading “0” current of DGTFET DRAM can reach to 1 nA/μm, which is much less than that of traditional 1T1C DRAM. And the RT of 2 s is far superior to the target value of 64 ms which is usually set to dynamic refresh time in computing system [21]. The RT of DGTFET DRAM is still larger than 300 ms when the temperature is increased to 85 °C, which authorizes its practicability in the harsh conditions. Furthermore, in DGTFET DRAM, the elimination of capacitor with larger size also exhibits its competitivity in high-density packaging. These advantages fully indicate that it is necessary to study DGTFET DRAM. Although these researches have demonstrated that DGTFET has the superior performance than conventional 1T1C DRAM, the results (RT and reading “0” current) are not optimal due to that fact that device configuration of DGTFET is not optimized.

In this paper, the spacer engineering of DGTFET is optimized to make DGTFET obtain the optimum performance. In TFETs, the spacer dielectrics have the strong influences on BTBT [22, 23]. In DGTFET, the spacers at the source and drain sides are closed to tunneling junctions, so they greatly affect the performance of DGTFET DRAM. This paper systematically analyzes the influences of spacer dielectric (low-k or high-k dielectrics) in each spacer on DGTFET DRAM and proposes an optimized spacer engineering. By the optimization of spacer engineering, the reading “0” current is depressed to 10^−14^A/μm, and RT can reach to 10 s. Finally, the scalability of DGTFET DRAM with the optimized spacer engineering is also discussed in this work.

## Device Structure and Simulation Method

**Fig. 1:**
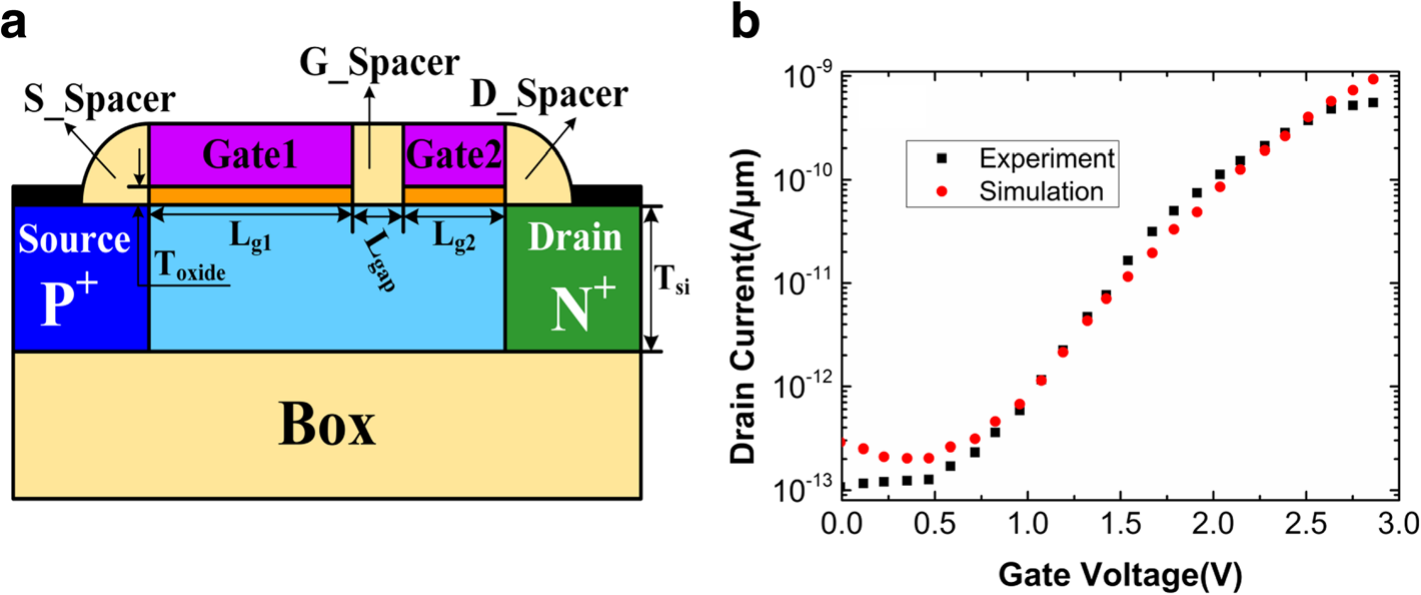
**a** Schematic of dual-gate TFET (DGTFET) DRAM cell. **b** Comparison between simulated transfer characteristic with experimental results for SOI TFET [25]

The DGTFET with a P-I-N configuration is illustrated in Fig. 1a. The source and drain regions are P^+^ doping (10^20^/cm^3^) and N^+^ doping (10^20^/cm^3^), respectively. The intrinsic channel region is divided into two parts: Gate1 region with the N^+^ polysilicon and Gate2 region with the P^+^ polysilicon. There is a short gap between the Gate1 and Gate2. The P^+^ polysilicon Gate2 can create as well as maintain the physical well for charge storage and replace the conventional TFET based DRAM that utilizes a P^+^ pocket region as the storage area. While for an N^+^ polysilicon Gate1, the hole concentration in underlap region between Gate1 and Gate2 is low, which is helpful for the reading operation. Thus, a P^+^ polysilicon Gate2 is opted to create a deeper storage region that could facilitate longer retention time, while an N^+^ polysilicon Gate1 is selected to control the tunneling mechanism during reading operation [20]. In Fig. 1a, the S_Spacer and D_Spacer refer to the spacers at the source and drain sides, respectively. The G_spacer refers to the spacer between the Gate1 and Gate2. The default material of the spacers is SiO_2_. The default device parameters are as follow: thickness of the silicon film (T_si_) is 20 nm, length of the Gate1 (L_g1_) is 400 nm, length of the Gate2 (L_g2_) is 200 nm, length of the gate gap (L_gap_) is 50 nm, and thickness of the Gate oxide HfO_2_ (T_oxide_) is 3 nm.

The simulations of the DGTFET DRAM are carried out in Silvaco Atlas using a nonlocal BTBT model. The nonlocal BTBT tunneling model takes into account the spatial variation of the energy bands and generation/recombination of the opposite carrier type [24]. The parameters of tunneling model are calibrated according to the experimental results of SOI TFET [25]. The electron and hole tunnel mass is adjusted to be 0.22m_0_ and 0.52m_0_, respectively, where m_0_ is the rest mass of electron. The simulated transfer characteristic of SOI TFET is consistent with experimental results, as shown in Fig. 1b, which authorizes the models applied in this paper. Due to the heavy doping in source and drain regions, the bandgap narrowing model and Fermi-Dirac statistics are also considered. Furthermore, the Shockley-Read-Hall recombination as well as doping and electric field-dependent mobility models are also applied. All the model parameters are consistent with those in [19, 20]. Because carrier lifetime governs the carrier generation/recombination during holding operation, it influences the RT of DGTFTET DRAM. According to the different carrier lifetimes varying in between 1 μs and 10 ns in [26–28], the electron and hole lifetimes are set to 100 ns. Scharfetter relation and Schenk models are used to include doping and temperature dependence of lifetime, respectively.

## Results and Discussion

### Operating Mechanism

**Fig. 2:**
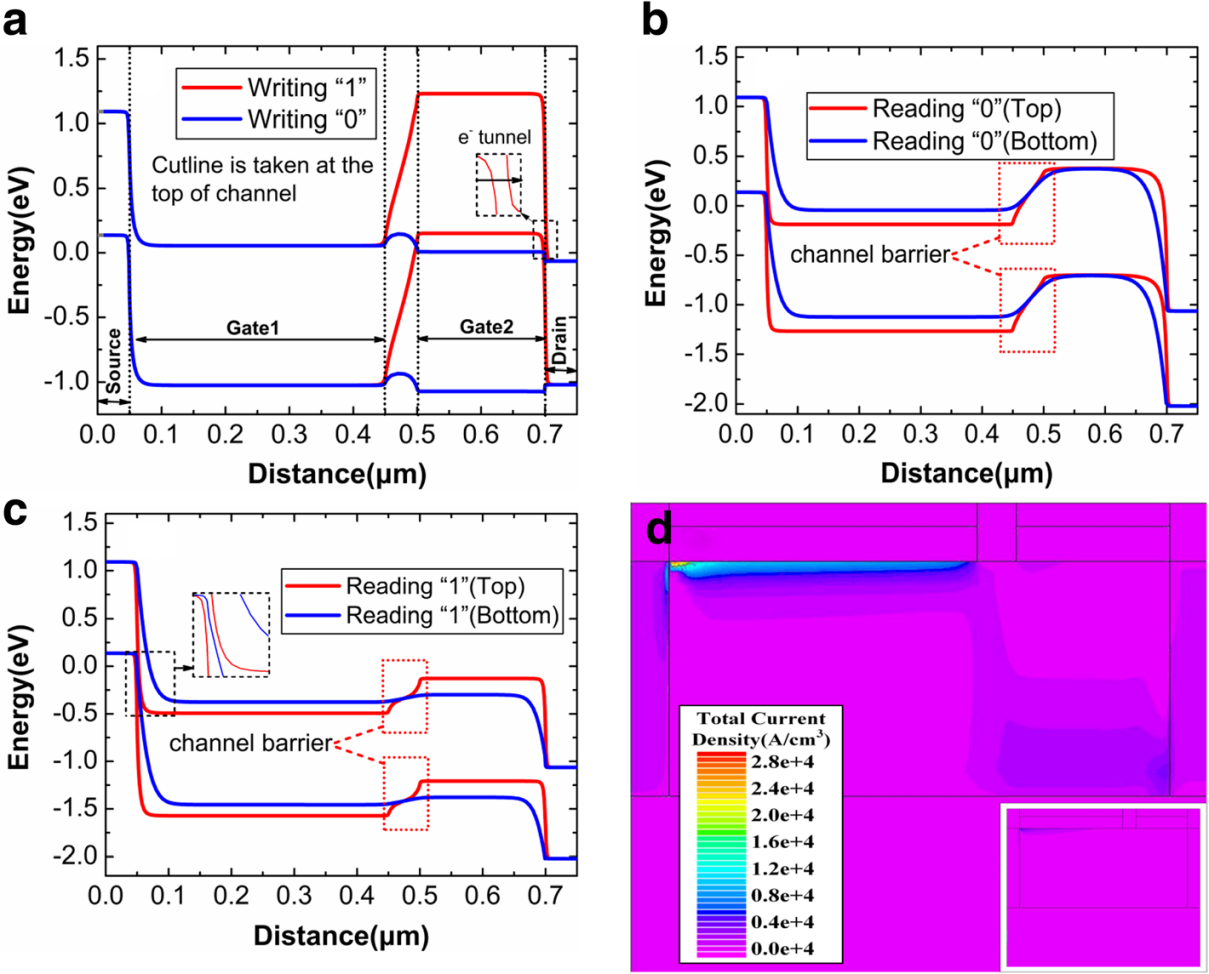
Energy bands from source to drain. **a** Energy bands after writing “1” with negative Gate2 bias and after writing “0” with positive Gate2 bias. **b** Energy bands at the top and bottom of channel after reading “1”. **c** Energy bands at the top and bottom of channel after reading “0”. **d** Total current density after reading “1”

**Fig. 3:**
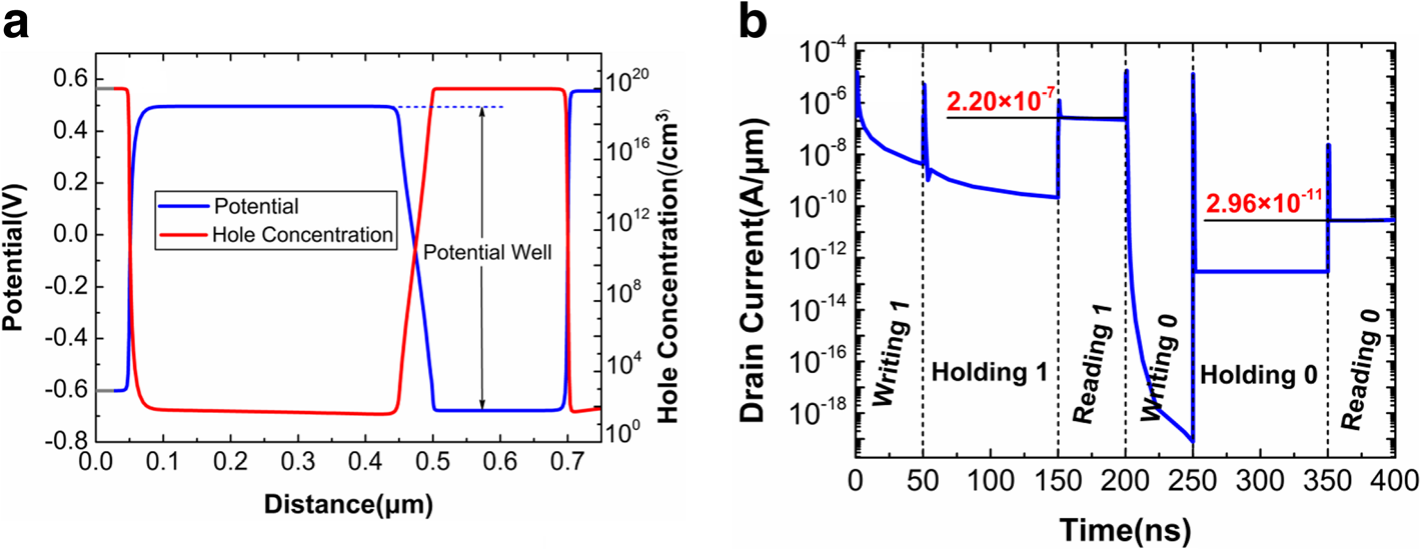
**a** Potential and hole concentration on the surface of channel after writing “1”. **b** Transient response of drain current for DGTFET DRAM operated in Table 1

In DGTFET DRAM, the writing and reading operations are controlled by BTBT at the drain and source tunneling junctions, respectively. Figure 2 shows the energy bands during the different operations. As shown in Fig. 2a, during the writing “1”, the Gate2 with a negative bias significantly puts up the energy band of channel under Gate2 so that an extremely small tunneling barrier is created at the drain side. Thus, the electrons tunnel towards the drain side and the holes are accumulated into the deep potential well (1.2 V), as shown in Fig. 3a. During the writing “0”, the Gate2 with a positive bias makes the holes expel from this potential well and recombined at the drain side [29]. Figure 2b, c shows the energy bands after reading “1” and “0”, respectively. Figure 2b illustrates that there is a channel barrier between the Gate1 and Gate2, but this does not exist at the bottom of channel. Besides, the tunneling distance at the source side is smaller at the top of channel. This demonstrates that an inclined conduction path (from front interface for Gate1 to back interface for Gate2) is formed during the reading “1”, which can also be demonstrated by the current density of Fig. 2d. During the reading “0”, the obvious channel barrier can be found in Fig. 2c, which restrains the reading “0” current. The inset of Fig. 2d shows that electrons tunneling from source region cannot cross this channel barrier to form the higher reading “0” current.

A proper programming condition is important for DGTFET DRAM. A larger negative bias should be applied at Gate2 so that the saturated BTBT is induced during writing “1”. During holding “1”, a small negative bias is applied at Gate2 to retain holes in potential well for a long time. For reading operations, the higher Gate1 bias strengthens the BTBT during reading “1”, but which is detrimental for reading “0” current. The Gate2 with the appropriate bias not only can enhance the reading “1” current but also can weaken the reading “0” current. Applying the optimized programming condition in Table 1, the transient response of DGTFET DRAM with the default parameters is shown in Fig. 3b. The writing and reading as well as holding times are set to 50 ns, and 100 ns, respectively. The obtained current ratio of reading “1” to reading “0” is about 10^4^, which is the same as that in [17, 19, 20].

**Table 1:** Optimized programming condition for DGTFET DRAM

Operation	V_g1_	V_g2_	V_d_	V_s_
Writing “1”	0 V	− 1.3 V	0 V	0 V
Writing “0”	0 V	1.3 V	0 V	0 V
Holding	0 V	− 0.2 V	0 V	0 V
Reading	0.7 V	0.7 V	1 V	0 V

### Impact of Spacer Dielectrics

In DGTFET DRAM, the usage of low-k or high-k dielectrics in three spacers (S_Spacer, G_Spacer and D_Spacer) will influence its performance. In this design, the low-k and high-k dielectrics choose the SiO_2_ and HfO_2_, respectively. If each spacer uses SiO_2_ or HfO_2_, there will be eight combinations of spacer engineering at all. For more comprehensive analysis, the performance properties of DGTFET DRAM with each combination, including reading “1” (*I*_1_) and “0” (*I*_0_) currents as well as current ratio (*I*_1_/ *I*_0_), are extracted from the transient responses, as shown in Table 2. In order to assess the RT, these parameters are also extracted when the holding time is increased to 2 s, which will be discussed in the following sections. In Table 2, the letters “S” and “H” represent SiO_2_ and HfO_2_, respectively, and three letters of each abbreviation respectively represent S_Spacer, G_Spacer, and D_Spacer.

**Table 2:** Extracted performance properties of DGTFET DRAM with different spacer dielectrics

	Holding time = 100 ns			Holding time = 2 s		
	I_1_(A/μm)	I_0_(A/μm)	I_1_/I_0_	I_1_(A/μm)	I_0_(A/μm)	I_1_/I_0_
S/S/S	2.20 × 10^−7^	2.96 × 10^−11^	7.45 × 10^3^	1.29 × 10^−7^	2.51 × 10^−8^	5.12
S/H/S	2.20 × 10^−7^	2.95 × 10^−11^	7.45 × 10^3^	1.29 × 10^−7^	2.38 × 10^−8^	5.43
S/S/H	2.02 × 10^−7^	1.40 × 10^−14^	1.44 × 10^7^	1.29 × 10^−7^	6.46 × 10^−12^	2.00 × 10^4^
S/H/H	2.01 × 10^−7^	1.35 × 10^−14^	1.49 × 10^7^	1.29 × 10^−7^	6.13 × 10^−12^	2.11 × 10^4^
H/S/S	1.29 × 10^−9^	2.81 × 10^−11^	4.58 × 10^1^	9.08 × 10^−14^	1.29 × 10^−11^	7.07 × 10^−3^
H/H/S	1.29 × 10^−9^	2.81 × 10^−11^	4.58 × 10^1^	1.53 × 10^−14^	1.29 × 10^−11^	1.19 × 10^−3^
H/S/H	3.77 × 10^−9^	1.21 × 10^−14^	3.11 × 10^5^	3.04 × 10^−13^	1.58 × 10^−14^	1.92 × 10^1^
H/H/H	3.81 × 10^−9^	1.21 × 10^−14^	3.15 × 10^5^	2.52 × 10^−13^	1.49 × 10^−14^	1.69 × 10^1^

From Table 2, the optimum spacer engineering can be selected. The *I*_1_ are about 10^−7^A/μm and 10^−9^A/μm when the SiO_2_ and HfO_2_ are used in S_Spacer, respectively. When the D_Spacer applies the HfO_2_, the *I*_0_ is low to about 10^−14^A/μm. Therefore, the optimum spacer configuration of DGTFET DRAM is that low-k and high-k dielectrics should be used at the source and drain sides. The specific reasons will be analyzed systematically in the following sections.

#### Impacts of S_Spacer Dielectric

**Fig. 4:**
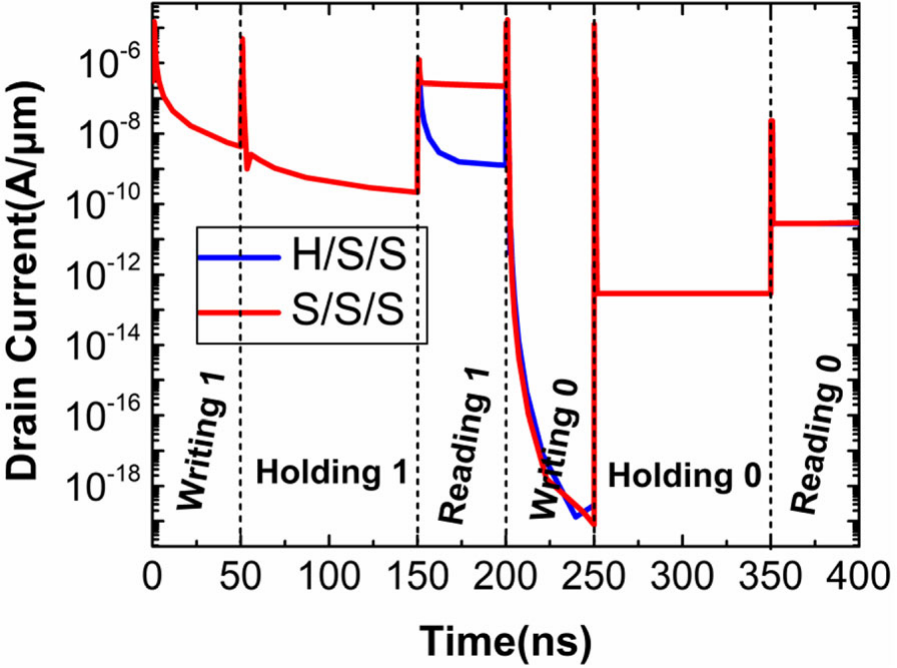
Transient responses of drain currents for H/S/S and S/S/S operated in Table 1

In order to analyze the influences of S_Spacer, the transient responses of drain currents for H/S/S and S/S/S are compared in Fig. 4. It can be observed that the reading “1” current is improved by about two orders of magnitude when the SiO_2_ is chosen as the S_Spacer dielectric.

**Fig. 5:**
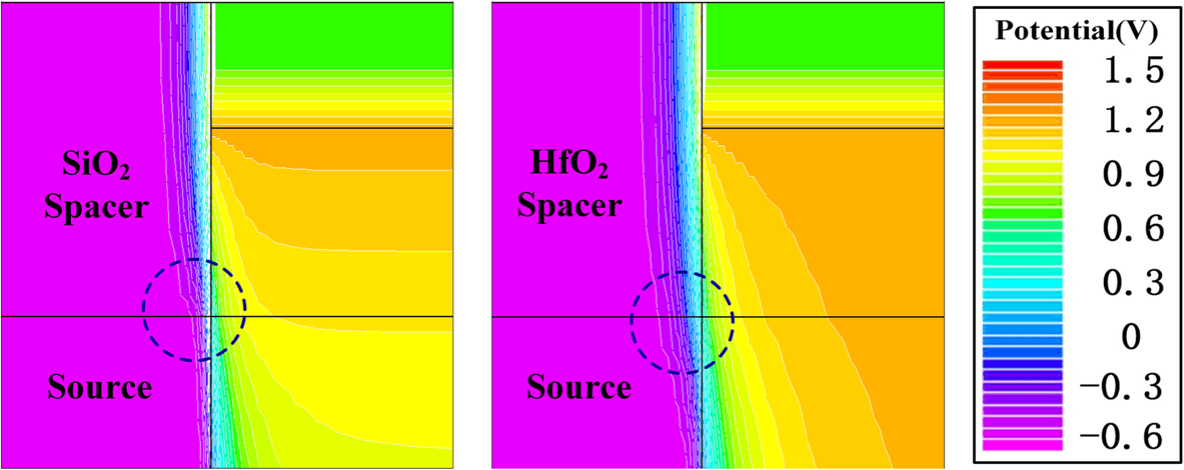
Potential contours of the S/S/S (left) and H/S/S (right) after reading “1”

**Fig. 6:**
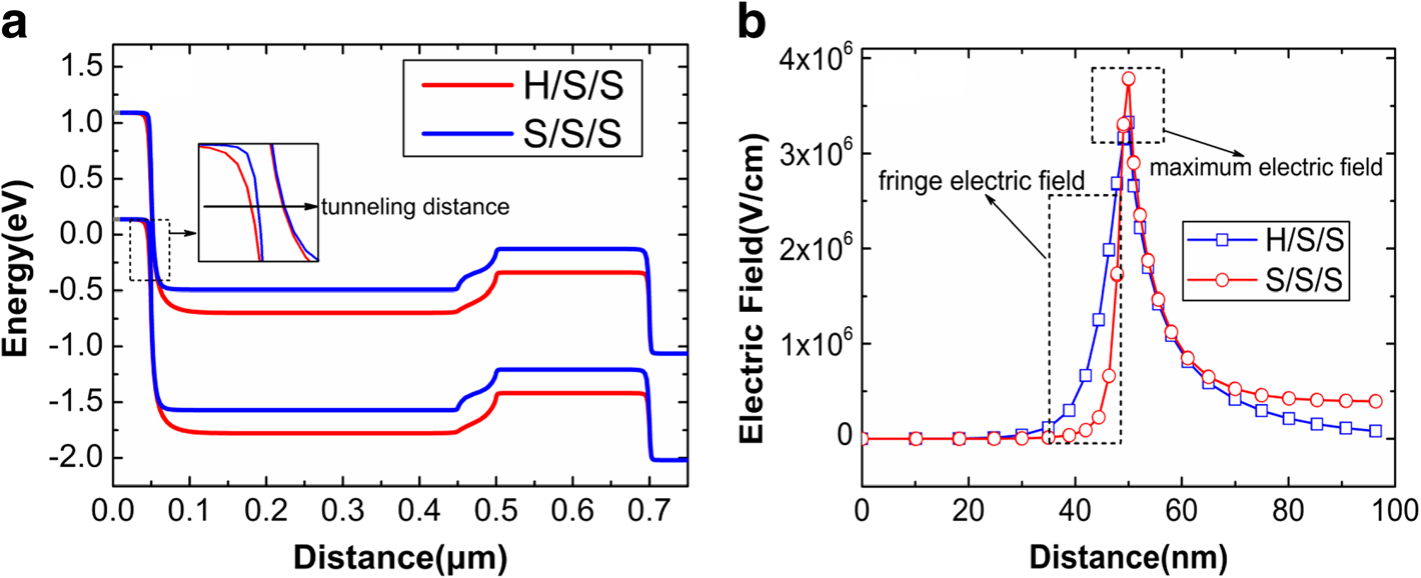
**a** Energy band from source to drain and **b** electric field at the top of source tunneling junction

Figure 5 shows the potential contours of S/S/S and H/S/S. At the source tunneling junction, the surface depletion region of H/S/S is extended obviously compared with that of S/S/S, as shown in the circle in Fig. 5. The extended surface depletion region increases the tunneling barrier width. Figure 6a shows the energy bands after reading “1”. As shown in local enlarged region of this figure, the tunneling distance (see the arrows) of H/S/S is obviously larger than that of the S/S/S, which is caused by the extended surface depletion region. Besides, after reading “1”, the electric field at the top of source tunneling junction is shown in Fig. 6b. It can be found that fringe electric field of H/S/S is larger than that of S/S/S, which is the main reason for the extension of surface depletion region. In a word, S_Spacer with high-k (HfO_2_) dielectric generates the larger fringe electric field so that the surface depletion region at source tunneling junction is extended, which increases the tunneling distance of electrons and decreases the reading “1” current. Furthermore, it can be also found from Fig. 6b that maximum electric field of S/S/S is larger than that of H/S/S. The exponential relation between BTBT rate and electric field makes the tunneling current of S/S/S much larger than that of H/S/S [30]. Therefore, the S_Spacer with the low-k dielectric (SiO_2_) is beneficial to the reading “1” current.

In Fig. 6a, the S_Spacer dielectric also brings the impact on the energy band of channel region. In Fig. 6b, the electric field of S/S/S is larger in channel region, so its channel potential is less than that of H/S/S. As a result, the higher energy level can be found in S/S/S. But this cannot bring effects on the tunneling barrier and reading “1” current.

#### Impacts of D_Spacer Dielectric

**Fig. 7:**
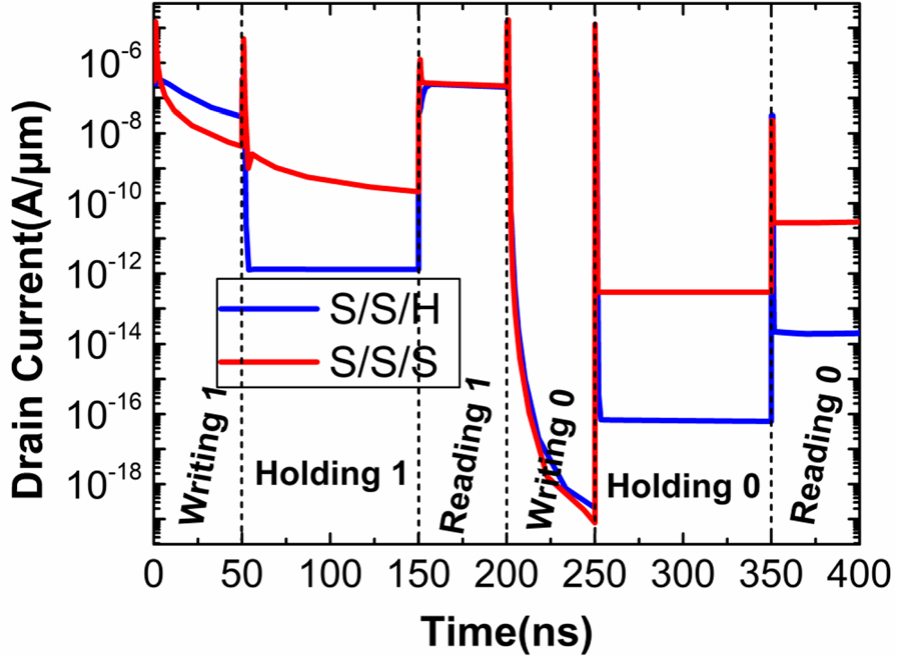
Transient responses of drain currents for S/S/H and S/S/S operated in Table 1

Subsequently, the D_Spacer is also investigated in this paper. Keeping the constant S_Spacer and G_Spacer (SiO_2_ is used in these two Spacers), the different transient drain currents caused by the different D_Spacer dielectrics are illustrated in Fig. 7. Apart from the reading “1”and writing “0”, the other operations have obvious dependence on D_Spacer dielectric. This is because that D_Spacer is far from the reading “1”conduction path (bottom of channel under the Gate2). It can be learned from the previous operating mechanism that writing and holding operations are governed by Gate2, so the D_Spacer dielectric can bring the influences on the these two operations.

During the holding operation, the holes accumulated during the writing “1” are removed from the potential well and recombined at drain side. So the holding “1” current mainly comes from the SRH recombination current. Due to the stronger controllability of Gate2 over the channel in S/S/S, S/S/S has the greater recombination rate and holding “1” current. But it is much less than BTBT current during reading “1”, so the higher holding “1” current cannot be reflected in reading “1” current.

**Fig. 8:**
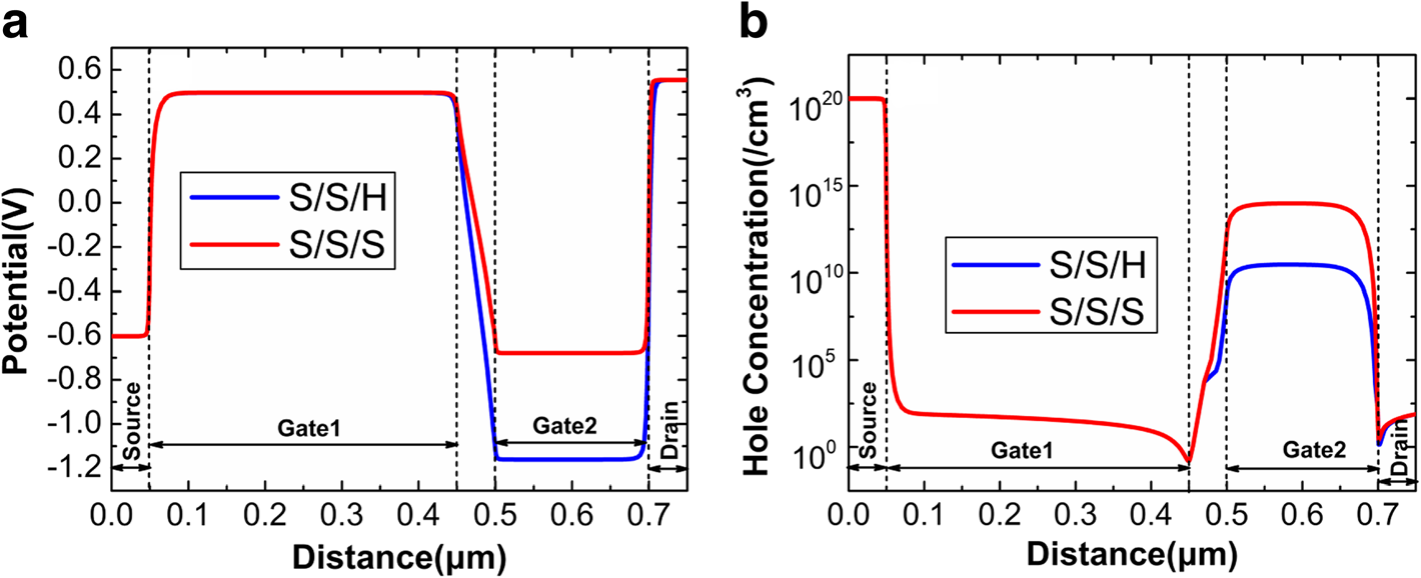
**a** Potential contour after writing “1”. **b** Hole concentration after holding “0”

During the writing “1”, the D_Spacer dielectric significantly influences the potential well depth. The potential contour in Fig. 8a shows that D_Spacer with HfO_2_ dielectric creates a deeper potential well. It implies that the effective BTBT between the drain and channel is extended into deeper channel region. Therefore, the writing “1” current of S/S/H is higher than that of S/S/S. During holding “0”, although a small negative bias (− 0.2 V) is applied at Gate2, it can also put up the energy band of channel under Gate2, which induces the BTBT at the drain side. Through the previous analysis, it can be learned that D_Spacer with SiO_2_ dielectric enhances the BTBT at the drain tunneling junction during holding “0”, which can be demonstrated by the higher hole concentration for S/S/S, as shown in Fig. 8b. Therefore, the D_Spacer with SiO_2_ dielectric results into the higher holding “0” current.

**Fig. 9:**
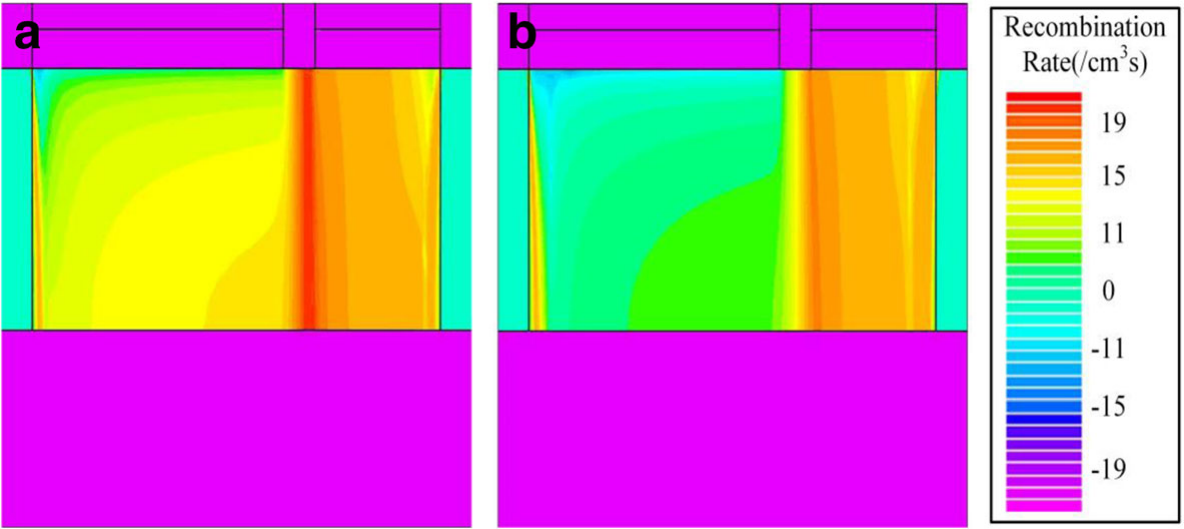
Recombination rate of **a** S/S/S and **b** S/S/H after reading “0”

During reading “0”, because the channel barrier between the Gate1 and Gate2 prevents the electrons flowing towards drain side, the difference of reading “0” current for S/S/H and S/S/S is mainly caused by the recombination current. The more holes are accumulated during the holding “0” for S/S/S, so the dropping energy band makes the recombination rate of S/S/S larger than that of S/H/S during reading “0”, as shown in Fig. 9. As a result, when the D_Spacer using SiO_2_, the higher reading “0” current should be attributed to the higher recombination current, which is caused by the more accumulated holes during holding “0”.

In summary, the optimum spacer engineering for DGTFET DRAM is that the spacers at the source and drain sides should apply the low-k and high-k dielectrics, respectively. It can be seen from Table 2 that G_Spacer has no influence on DGTFET DRAM when the other spacers keep unchanged. This is because that BTBTs dominating the writing and reading operations are free from the influence of G_Spacer.

### Retention Time

As is explained previously, the hole recombination and generation during holding “1” and “0” degrade the state “1” and “0”, respectively. As a result, it is necessary to study the performance degradation of DGTFET DRAM at the longer holding time. In Table 2, the *I*_1_ and *I*_0_ degrade greatly with the increasing of holding time. In all the devices, *I*_1_/*I*_0_ is still larger than 10^4^ for device with optimum spacer engineering (S/S/H and S/H/H) when the holding time rises to 2 s.

**Fig. 10:**
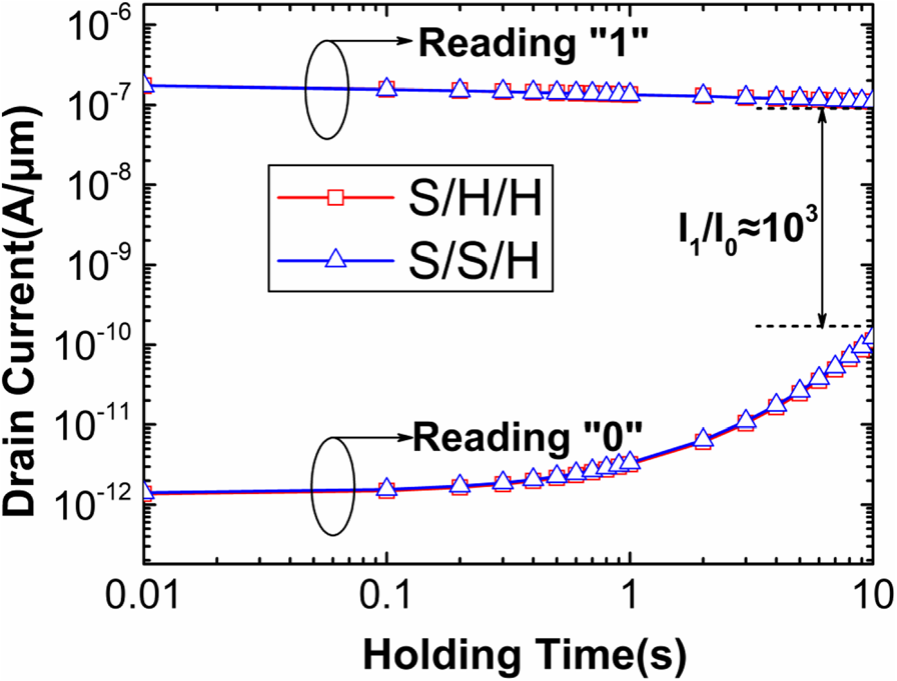
Variation of reading current with the holding time for S/S/H and S/H/H

Generally, the holding time required to reduce the maximum sense margin (difference between *I*_1_ and *I*_0_) by 50% is assessed as RT. In this design, a stricter RT is defined as the maximum holding time when the *I*_1_/*I*_0_ is higher than 10^3^. Figure 10 shows the variation of reading current with the holding time for S/S/H and S/H/H. It can be found that the current ratio of S/H/S and S/H/H is as high as 10^3^ when the holding time rises to 10 s. As a result, the RT of DGTFET DRAM with optimum spacer engineering can reach to 10 s. This is far higher than target value of 64 ms. Table 3 compares the performance properties in this work with that in [17–20]. In [19, 20], the current ratio is only 10^2^, and the RT is much smaller than 10 s. Furthermore, the reading “0” current in this work is two orders of magnitude lower than that in [19, 20]. The experiment results about FD-SOI TFET DRAM also shows that their reading “0” current and RT are inferior than that in this work. This superior performance shows that DGTFET is a substitute for low power DRAM. The optimization of spacer engineering makes the DGTFET DRAM obtain low reading “0” current and high RT, which is helpful for the reduction of static and dynamic power consumption.

**Table 3:** Performance properties of various TFET DRAM utilized as DRAM

Device configuration [Reference]	Device size	RT	Reading “0” current
FD-SOI TFET with intrinsic region [17]	*L*_g_ 400 nm; Intrinsic Region Length (*L*_in_) 200 nm		1.2 μA/μm
DG FD-SOI TFET [18]	*L*_g1_ 400 nm; *L*_g2_ 200 nm; *L*_in_ 200 nm	100 μs~#ms	50 nA/μm
DGTFET with front gate [19]	*L*_g1_ 400 nm; *L*_g2_ 200 nm; *L*_gap_ 200 nm	1.5 s	0.1 nA/μm
DGTFET with back gate [20]	*L*_front gate_ 400 nm; *L*_back gate_ 200 nm	170 ms	0.1 nA/μm
Prsent work	*L*_g1_ 400 nm; *L*_g2_ 200 nm; *L*_gap_ 50 nm	10 s	14fA/μm

### Scalability of DGTFET DRAM

Although the DGTFET DRAM solves the problem of memory cell density due to the elimination of capacitor with the large size, it is necessary to study its scalability. The goal of scalability is to keep RT higher than 2 s. Table 4 extracts the performance properties of S/S/H with the decreasing of *L*_g1_, *L*_g2_, and *L*_gap_. In Table 4, the three numbers of each abbreviation represent *L*_g1_, *L*_gap_, and *L*_g2_.

**Table 4:** Extracted performance properties of S/S/H with the decreasing of device size

	Holding time = 100 ns			Holding time = 2 s		
	*I*_1_(A/μm)	*I*_0_(A/μm)	*I*_1_/*I*_0_	*I*_1_(A/μm)	*I*_0_(A/μm)	*I*_1_/*I*_0_(A/μm)
400/50/200	2.02 × 10^−7^	1.40 × 10^−14^	1.44 × 10^7^	1.29 × 10^−7^	6.46 × 10^−12^	2.00 × 10^4^
300/50/200	1.78 × 10^−7^	8.33 × 10^−12^	2.13 × 10^4^	1.09 × 10^−7^	3.67 × 10^−11^	2.97 × 10^3^
200/50/200	1.28 × 10^−7^	5.23 × 10^−11^	2.45 × 10^3^	7.99 × 10^−8^	2.26 × 10^−10^	3.54 × 10^2^
100/50/200	4.45 × 10^− 8^	3.06 × 10^− 10^	1.45 × 10^2^	3.24 × 10^− 8^	1.17 × 10^−9^	2.76 × 10^1^
200/50/150	1.36 × 10^−7^	8.17 × 10^−11^	1.67 × 10^3^	8.01 × 10^−8^	5.56 × 10^−10^	1.44 × 10^2^
200/50/100	1.47 × 10^−7^	2.41 × 10^−10^	6.08 × 10^2^	8.22 × 10^−8^	2.51 × 10^−9^	3.28 × 10^1^
200/40/150	1.36 × 10^−7^	8.22 × 10^−11^	1.65 × 10^3^	8.10 × 10^−8^	5.57 × 10^10^	1.45 × 10^2^
200/30/150	1.35 × 10^−7^	9.00 × 10^−11^	1.51 × 10^3^	8.11 × 10^−8^	5.82 × 10^−10^	1.39 × 10^2^
200/20/150	1.35 × 10^−7^	9.54 × 10^−11^	1.41 × 10^3^	8.08 × 10^−8^	6.24 × 10^−10^	1.29 × 10^2^
200/10/150	1.35 × 10^−7^	1.02 × 10^−10^	1.32 × 10^3^	8.11 × 10^−8^	1.00 × 10^−9^	8.10 × 10^1^

From Table 4, it can be observed that *I*_1_ extremely decreases when the L_g1_ reduces to 100 nm from 200 nm. The scaling of L_g1_ beyond 100 nm narrows the n-type-induced barrier, resulting into the reduced gate controllability and degraded *I*_1_. The *I*_0_ significantly increases with the decreasing of *L*_g1_ and *L*_g2_. The reduction of *L*_g2_ decreases the channel barrier width between Gate1 and Gate2, which promotes a part of electrons to cross the barrier to form the higher *I*_0_. In addition, the continuous scaling down of *L*_g1_ weakens the ability of Gate1 to restrict the tunneling electrons on the surface of channel during reading “0”. In Table 4, the *L*_gap_ has no obvious influence on the *I*_1_, but the *I*_0_ slightly increases with the decreasing of the *L*_gap_. Reducing *L*_gap_ below 20 nm permits a higher tunneling towards Gate2, thereby degrading state “0”, thus, reducing retention time.

In order to ensure that the *I*_1_/*I*_0_ and retention time are larger than 10^2^ and 2 s, respectively, the minimum *L*_g1_, *L*_g2_, and *L*_gap_ are regard as 200, 150, and 20 nm, respectively. This minimum device size is slightly smaller than that in [17–20], as shown in Table 3. However, the minimum size of DGTFET DRAM is still larger than that of 20 nm/18 nm node 1T1C DRAM [31], which is the inherent shortcoming for DGTFET DRAM. But its advantages of capacitor-less, low power, and high RT cannot be ignored under the help of optimization of spacer engineering. Reducing the size of DGTFET DRAM beyond 100 nm will be the focus of our work in the future.

## Conclusions

In this paper, the optimization of spacer engineering for DGTFET DRAM is studied by Silvaco-Atlas tool. The spacers at the source and drain sides have the main influences on performance of DGTFET DRAM. The enlarged fringe electric field by the source spacer with HfO_2_ makes the surface depletion region extended at source tunneling junction, which decreases the reading “1” current. When the SiO_2_ dielectric is used in drain spacer, the stronger BTBT induces more holes during holding “0”, which enhances the recombination current during reading “0”. Therefore, the optimum spacer engineering is that low-k and high-k dielectrics should be used in drain and source spacers, respectively. Through the optimization of spacer engineering, the DGTFET DRAM obtains prominent advantages—extremely low reading “0” current and higher retention time (10s) comparing to other related works. In addition, the analysis about scalability reveals that its minimum device size is still larger than that in latest 20 nm/18 nm node 1T1C DRAM. This inherent shortcoming indicates that reducing the size of DGTFET DRAM beyond 100 nm will be the focus of our work for DGTFET DRAM in the future.

## Abbreviations

DGTFET: Dual-gate tunneling field effect transistor
DRAM: Dynamic random access memory

## References

[1] Sunami H, Kure T, Yagi K, Wada Y, Yamaguchi K, Miyazawa H (1985). Scaling considerations and dielectric breakdown improvement of a corrugated capacitor cell for a future DRAM. IEEE J Solid-St Circ.

[2] Mandelman JA, Dennard RH, Bronner GB, DeBrosse JK, Divakaruni R, Li Y (2002). Challenges and future directions for the scaling of dynamic random-access memory (DRAM). IBM J Res Dev.

[3] Mueller W, Aichmayr G, Bergner W, Erben E, Hecht T, Kapteyn C (2005). Challenges for the DRAM cell scaling to 40nm.

[4] Kim SK, Choi GJ, Lee SY, Seo M, Lee SW, Han JH (2008). Al-doped TiO2 films with ultralow leakage currents for next generation DRAM capacitors. Adv Mater.

[5] Lee SY, Chang J, Choi J, Kim Y, Lim HJ, Jeon H (2017). Investigation of ultrathin Pt/ZrO2-Al2O3-ZrO2/TiN DRAM capacitors Schottky barrier height by internal photoemission spectroscopy. Curr Appl Phys.

[6] Tack M, Gao M (1990). The multistable charge-controlled memory effect in SOI MOS transistors at low temperatures. IEEE Trans. Electron Devices..

[7] Wann HJ, Hu C (1993) A capacitorless DRAM cell on SOI substrate. Proc IEEE IEDM:635–638.

[8] Okhonin S, Nagoga M, Sallese J, Fazan P (2001). A SOI capacitorless 1T–DRAM concept.

[9] Okhonin S, Nagoga M, Carman E, Beffa R, Faraoni E (2007). New generation of Z-RAM.

[10] Yoshida E, Tanaka T (2006). A capacitorless 1T-DRAM technology using gate-induced drain-leakage (GIDL) current for low-power and highspeed embedded memory. IEEE Trans. Electron Devices..

[11] Tanaka T, Yoshida E, Miyashita T (2004). Scalability study on a capacitorless 1T DRAM: from single-gate PD-SOI to double-gate Fin DRAM.

[12] Wang PF, Hilsenbeck K, Nirschl T, Oswald M, Stepper C, Weis M (2004). Complementary tunneling transistor for low power application. Solid State Electron.

[13] Li W, Liu HX, Wang SL, Chen SP (2017). Design of high performance Si/SiGe heterojunction tunneling FETs with a T-shaped gate. Nanoscale Res Lett.

[14] Ionescu M, Riel H (2011). Tunnel field-effect transistors as energy-efficient electronic switches. Nature.

[15] Zang SG, Liu XY, Lin X, Liu L, Liu W, Zhang DW et al (2010) Applications of tunneling fet in memory devices. IEEE, pp 1238–1240. http://ieeexplore.ieee.org/document/5667617/.

[16] Kim H, Park BG (2016). A 1T-DRAM cell based on a tunnel field-effect transistor with highly-scalable pillar and surrounding gate structure. J Korean Phys Soc.

[17] Biswas A, Dagtekin N, Grabinski W, Bazigos A, Royer CL, Hartmann JM (2014). Investigation of tunnel field-effect transistors as a capacitor-less memory cell. Appl Phys Lett.

[18] Biswas A, Ionescu AM (2015). 1T capacitor-less DRAM cell based on asymmetric tunnel FET design. IEEE J Electron Devi.

[19] Navlakha N, Lin JT, Kranti A (2016). Improved retention time in twin gate 1T DRAM with tunneling based read mechanism. IEEE Electron Device Lett.

[20] Navlakha N, Lin JT, Kranti A (2016). Improving retention time in tunnel field effect transistor based dynamic memory by back gate engineering. J Appl Phys.

[21] Almeida LM, Sasaki KRA, Caillat C, Aoulaiche M, Collaert N, Jurczak M (2013). Optimizing the front and back biases for the best sense margin and retention time in UTBOX FBRAM. Solid State Electron..

[22] Yoon YJ, Seo JH, Cho S, Kwon H, Lee JH, Kang M (2016). Effects of dual-spacer dielectrics on low-power and high-speed performance of sub-10 nm tunneling field-effect transistors. Jpn J Appl Phys.

[23] Mallik A, Chattopadhyay A, Guin S, Karmakar A (2013). Impact of a spacer–drain overlap on the characteristics of a silicon tunnel field-effect transistor based on vertical tunneling. IEEE Trans. Electron Devices..

[24] SILVACO International, Santa Clara, CA 95054, USA (2012). ATHENA/ATLAS User’s manual.

[25] Biswas DSS, Royer CL, Grabinski W, Ionescu AM (2012). TCAD simulation of SOI TFETs and calibration of non-local band to-band tunneling model. Microelectron Eng.

[26] Kim S, Choi SJ, Moon DI, Choi YK (2012). Carrier lifetime engineering for floating-body cell memory. IEEE Trans. Electron Devices..

[27] Wan J, Royer CL, Zaslavsky A, Cristoloveanu S (2013). Progress in Z^2^-FET 1T-DRAM: retention time, writing modes, selective array operation, and dual bit storage. Solid State Electron..

[28] Rodriguez N, Cristoloveanu S, Gamiz F (2011). Novel capacitorless 1T-DRAM cell for 22-nm node compatible with bulk and SOI substrates. IEEE Trans. Electron Devices..

[29] Munteanu D, Weiser DA, Cristoloveanu S, Faynot O, Pelloie JL, Fossum JG (1998). Generation–recombination transient effects in partially depleted SOI transistors: systematic experiments and simulations. IEEE Trans Electron Devices.

[30] Kane EO (1961). Theory of tunneling. J Appl Phys.

[31] Park JM, Hwang YS, Kim SW, Han SY, Park JS, Kim J (2015). 20nm DRAM: a new beginning of another revolution.

